# Effective Platform for the Production of Recombinant Outer Membrane Vesicles in Gram-Negative Bacteria

**DOI:** 10.4014/jmb.2003.03023

**Published:** 2020-05-31

**Authors:** Anthicha Kunjantarachot, Teva Phanaksri

**Affiliations:** Chulabhorn International College of Medicine, Thammasat University, Pathumthani 12120, Thailand

**Keywords:** Recombinant outer membrane vesicles, gram-negative bacteria, lipoprotein signal peptide, platform

## Abstract

Bacterial outer membrane vesicles (OMVs) typically contain multiple immunogenic molecules that include antigenic proteins, making them good candidates for vaccine development. In animal models, vaccination with OMVs has been shown to confer protective immune responses against many bacterial diseases. It is possible to genetically introduce heterologous protein antigens to the bacterial host that can then be produced and relocated to reside within the OMVs by means of the host secretion mechanisms. Accordingly, in this study we sought to develop a novel platform for recombinant OMV (rOMV) production in the widely used bacterial expression host species, *Escherichia coli*. Three different lipoprotein signal peptides including their Lol signals and tether sequences—from *Neisseria meningitidis* fHbp, *Leptospira interrogans* LipL32, and *Campylobactor jejuni* JlpA—were combined upstream to the GFPmut2 model protein, resulting in three recombinant plasmids. Pilot expression studies showed that the fusion between fHbp and GFPmut2 was the only promising construct; therefore, we used this construct for large-scale expression. After inducing recombinant protein expression, the nanovesicles were harvested from cell-free culture media by ultrafiltration and ultracentrifugation. Transmission electron microscopy demonstrated that the obtained rOMVs were closed, circular single-membrane particles, 20–200 nm in size. Western blotting confirmed the presence of GFPmut2 in the isolated vesicles. Collectively, although this is a non-optimized, proof-of-concept study, it demonstrates the feasibility of this platform in directing target proteins into the vesicles for OMV-based vaccine development.

## Introduction

Outer membrane vesicles (OMVs) are closed, spherical lipid bilayer structures that are released naturally and constitutively from gram-negative bacteria during their growth. These nanostructures play significant roles in many physiological and pathological processes of the bacteria that correspond to cell proliferation and survival [1, 2]. Pathogenic bacteria use OMVs as a secretion and delivery system to disseminate toxins, virulence factors, and other active biomolecules to host cells to help carry out host cell invasion and initiate the infection [3]. Since OMVs are derived from bacterial membranes, they are enriched in elements that are similar to those presented in the outer membranes of their parent cells, such as outer membrane proteins, antigens, virulence factors, toxins, and lipids, as well as inner membrane and periplasmic components [4]. Upon binding to host cells, the delivery of antigens, virulence factors, and other immunomodulatory molecules to the cells not only initiates the infection but also induces an inflammatory cascade and protective immune responses against the pathogens [3].

Owing to their unique characteristics and the presence of intrinsic immunostimulatory components, these nanovesicles can potentially be used as vaccine antigens and adjuvants. OMV-based vaccines have been developed and tested for their efficacy against many bacterial diseases [5]. The meningococcal group B OMV vaccine is an explicit example of using bacterial OMVs as a vaccine antigen. It is currently the only FDA-approved OMV-based vaccine available on the market (known as Bexsero) and includes recombinant neisserial antigens in its formulation [6]. This vaccine preparation was able to generate specific immune responses towards the antigens and conferred greater than 70% protection [7]. OMV-based vaccines are superior to their whole-cell or attenuated counterparts in terms of vaccine safety because these subcellular nanoparticles are noninfectious and non-replicable. Most OMVs that have thus far been tested in vaccine efficacy studies (either in vitro or in vivo) are derived from the isolation of homologous vesicles from corresponding bacterial species through various methods [8]. These methods include direct isolation of the vesicles from cell-free culture media, yielding natural spontaneously released OMVs (sOMVs) as well as detergent-dependent and detergent-free extractions of OMVs from concentrated cells, yielding detergent-derived OMVs (dOMVs) and native OMVs (nOMVs), respectively [9]. Depending on the production method, biomaterials incorporated into the OMVs can vary in their protein and lipid compositions. In addition, the composition of OMVs can be affected by the growth phase, media components, or a specific stress signal [10, 11].

While homologous OMVs are increasingly attractive in the field of vaccine development, their production has some drawbacks; for example, productivity and the biocomposition of the isolated vesicles vary among growth conditions and depend on several factors [12, 13]. Therefore, the number of antigens and immunogenic molecules present within the vesicles can be inconsistent. Furthermore, some bacterial species require special media or growth conditions, which could adversely affect the production process, time, and cost for future clinical use. To overcome these limitations, recombinant OMVs (rOMVs) have been developed. Biotechnological advancements have made it possible to create OMVs that are decorated with heterologous antigenic protein(s) of interest in bacterial host species. By constructing a protein expression vector that contains the desired sequence along with the proper leading signal and introducing that recombinant vector into bacterial host cells, heterologous target proteins can be produced and displayed on the outer membrane and released as part of the rOMVs, which can subsequently be isolated from the culture media. As yet, there is no effective method that uses bacterial host systems to generate rOMVs with the incorporation of heterologous proteins. Therefore, the aim of this study was to establish the technique and develop a prototype platform for the production of rOMVs in an *Escherichia coli* (*E. coli*) host: a host cell that could recognize the heterologous signal peptide fused to the gene of interest, green *fluorescent protein mutant2* (*gfpmut2*), express and relocate the target protein (GFPmut2) to its outer membrane and subsequently into vesicles. In future applications, this platform could be used to make OMV vaccine candidates containing antigenic proteins of interest that are not only bacterial-derived—they could be from any pathogen. Moreover, due to the intrinsic adjuvant properties of OMVs, they could also be alternatively utilized as a vaccine adjuvant or a delivery vehicle to carry proteins of interest to the target cells.

## Materials and Methods

### Bacterial Strains

*E. coli* strain DH5α was used for plasmid propagation and isolation. Recombinant protein expression, isolation, and purification of rOMVs carrying the protein of interest were conducted in *E. coli* strain Lemo21(DE3) (New England BioLabs Inc., USA).

### Production of Heterologous *gfpmut2* Sequence Combined with Bacterial Type II or Lipoprotein Signal Peptide

N-terminal sequences that included the signal peptide of three bacterial lipoproteins were selected and fused with the mature sequence of *gfpmut2*. The three lipoproteins were: (1) factor H-binding protein (fHbp) of *Neisseria meningitidis* (accession number: ACS92735.1) [14], a surfaced-exposed lipoprotein that acts as a bacterial virulence factor; (2) subsurface leptospiral major outer membrane lipoprotein LipL32 of *Leptospira interrogans* serovar Lai strain 55601 (accession number: WP_000736494.1) [15]; and (3) JlpA protein of *Campylobacter jejuni* (accession number: WP_009882608.1), a surface-exposed lipoprotein that promotes adherence to host epithelial cells [16]. The full-length double-stranded *gfpmut2* DNA sequence combined with the lipoprotein signal peptides derived from either fHbp, LipL32, or JlpA upstream to its 5′ end were optimized, chemically synthesized, and validated by the GeneArt® Gene Synthesis Service for Strings DNA fragments (Invitrogen, USA). These DNA fragments were generally referred to as *lipo-gfpmut2*.

### Construction of Recombinant Plasmid Expressing GFPmut2

Full-length heterologous *lipo-gfpmut2* fragments containing N-terminal portions of fHbp, LipL32, and JlpA are annotated as *lipo-fHpb-gfpmut2*, *lipo-lipL32-gfpmut2*, and *lipo-jlpA-gfpmut2*, respectively. Each synthetic gene construct was resuspended in DNase/RNase-free water and kept at -20°C until use. For the expression of GFPmut2 protein, and hence the production of rOMVs, pET-22b plasmid DNA (Merck, Germany) was used as a backbone.

To create recombinant plasmid expressing GFPmut2, double digestion of the *lipo-gfpmut2* fragments and pET-22b plasmids with NdeI and XhoI restriction enzymes (New England BioLabs Inc.) was performed. Plasmid DNA from the digestion reaction was then subjected to 0.8% agarose gel electrophoresis to verify whether it was linearized. Double-digested, linearized plasmid DNA was then extracted from the gel, purified, and stored at -20°C until use. Following the double digestion, each *lipo-gfpmut2* fragment was ligated with linearized pET-22b vector using T4 DNA ligase (New England BioLabs Inc.) to yield circular recombinant plasmids.

Transformation of the recombinant pET-22b plasmids into *E. coli* strain DH5α host cells was accomplished by mixing the ligation reaction products with competent cells. Transfer of the recombinant plasmids into the bacteria was induced by heat shock at 42°C. After the addition of SOC medium, transformed bacteria were grown at 37°C and then plated on Luria–Bertani (LB) agar containing 50 μg/ml ampicillin for colony selection. The agar plates were incubated overnight at 37°C. Colony polymerase chain reaction (PCR) was conducted to screen for colonies that carried the recombinant plasmids, *i.e.*, positive transformants. Positive clones were then propagated, and recombinant plasmid DNA was extracted using a Presto Mini Plasmid Kit (Geneaid, Taiwan). The purified plasmids were submitted to a sequencing service (Bioneer, Republic of Korea) to validate the sequence of the *lipo-gfpmut2* inserts. The presence of a conserved lipobox in each validated heterologous fragment was subsequently verified using the protein sorting prediction software SignalP 5.0 server [17], available at https://services.healthtech.dtu.dk/service.php?SignalP-5.0, accessed February 28, 2020.

Following sequence validation, the recombinant pET-22b plasmids carrying *lipo-gfpmut2* were transformed into *E. coli* Lemo21(DE3) host cells for the production of recombinant GPFmut2. A 25-ng aliquot of recombinant plasmid was mixed with the cells, which were then exposed to heat shock at exactly 42°C to allow the plasmids to enter the cells. After adding SOC medium, transformation cultures were plated on LB agar containing 100 μg/ml ampicillin and 30 μg/ml chloramphenicol (LB agar+ampicillin_100_+chloramphenicol_30_). The plates were incubated overnight at 37°C. Colonies able to grow on the selection media were used to inoculate the same preparation of liquid media to prepare a starter culture for the protein expression studies.

### Expression of Recombinant GFPmut2

A small-scale pilot protein expression study was initially completed to explore whether recombinant GFPmut2 was produced in the Lemo21(DE3) cells and, if so, to identify the optimal conditions for protein expression that yielded the highest level of protein production. The concentration of L-rhamnose, an inducer of T7 lysozyme production, was varied in the pilot expression studies, while the concentration of isopropyl β-D-1-thiogalactopyranoside (IPTG) was kept constant throughout the experiments.

Forty-five milliliters of LB broth+ampicillin_100_+chloramphenicol_30_ was inoculated with 1 ml of freshly grown Lemo21(DE3) starter culture harboring recombinant plasmids and was cultured at 30°C until the optical density at 600 nm (OD_600_) reached 0.4–0.8 (mid-log phase). The original culture was then divided into four 10-ml cultures: one for the uninduced control and the other three for recombinant GFPmut2 production in which the expression was induced by adding 40 μl of 0.1 M IPTG (final concentration 400 μM) and varying the final L-rhamnose concentration (500, 1,000, or 2,000 μM). All cultures were maintained at 30°C overnight. The following morning, the cultures were centrifuged at 6,000 ×*g* for 15 min at 4°C to collect the cell pellets. For the preliminary detection of GFPmut2 after induction, whole-cell lysate proteins from each culture were subjected to 12% sodium dodecyl sulfate polyacrylamide gel electrophoresis (SDS-PAGE) and compared with Precision Plus Protein Dual Color Standards (Bio-Rad, USA). The protein bands were visualized by staining the gels with Coomassie blue staining solution. To confirm that the expressed protein was functional, whole-cell lysates from different L-rhamnose induction concentrations were imaged under a fluorescence microscope (Delta Vision Elite Cell Imaging System, GE Healthcare Life Sciences, USA). After identifying the principle plasmid construct and determining the optimum culture conditions that enabled maximum GFPmut2 expression, large-scale culturing was carried out to increase the expression yield.

For the large-scale recombinant protein production, 500 ml of LB broth+ampicillin_100_+chloramphenicol_30_ was inoculated with 5 ml of freshly grown starter culture (1:10 ratio). When the OD_600_ reached 0.4–0.8, the optimal concentration of L-rhamnose (as determined in the pilot study) and 400 μM IPTG were applied to induce recombinant GFPmut2 expression. The culture was then grown at 30°C overnight. On the following day, the cells were separated from the culture media by centrifugation at 6,000 ×*g* for 15 min at 4°C. The medium was re-centrifuged with the same condition and then kept on ice or at -20°C until use.

### Isolation and Purification of rOMVs

Isolation of the rOMVs carrying GFPmut2 was achieved following a previously described protocol [18] with minor modifications. The supernatant fraction from the previous centrifugation step was filtered through a Rapid-Flow sterile disposable filter unit made of polyethersulfone (PES) with 0.45 μm pore size (Thermo Scientific, USA). The culture filtrate was then concentrated by ultrafiltration (centrifugation at 5,000 ×*g*, 4°C) using a Macrosep Advance centrifugal device with a 100K molecular weight cutoff PES membrane (Pall Corporation, USA). The rOMVs were pelleted from the concentrated culture filtrate via ultracentrifugation at 150,000 ×*g* at 4°C for 3 h in an Optima XE-100 ultracentrifuge (Beckman Coulter, USA). The pelleted OMVs were resuspended in 900 μl of phosphate-buffered saline (PBS), pH 7.4, and stored at -20°C.

### Detection and Characterization of Heterologous GFPmut2 Incorporated into the OMVs

The protein concentrations of the culture filtrate, concentrated culture filtrate, supernatant from the ultracentrifugation step, and the resuspended OMV solution were measured with a DC protein assay following the manufacturer’s microplate assay protocol (Bio-Rad) using bovine serum albumin as the protein standard. The resuspended OMV solution and the aforementioned samples and whole-cell lysate from the pilot study were equally loaded (approximately 35 μg/well) and subjected to 12% SDS-PAGE to detect the presence of target protein in the rOMVs. Proteins in the gels were visualized by Coomassie blue staining and the putative size of the target protein was compared with the Precision Plus Protein Prestained Dual Color Standard (Bio-Rad). To confirm the incorporation of polyhistidine-tagged recombinant GFPmut2 within the OMVs, the separated proteins were analyzed by Western blot. The proteins were transferred from the gel to a membrane and mouse anti-6xHis monoclonal antibody (mAb) [1:5,000 diluted in 5% skim milk in 0.2% Tris-buffered saline with Tween-20 (TBST); R&D Systems, Inc., USA] was used as the primary antibody to label the recombinant GFPmut2, which contained six histidine molecules at its C-terminus. The membrane was then incubated with rabbit anti-mouse IgG conjugated to horseradish peroxidase (1:10,000 diluted in 5% skim milk in 0.2% TBST; Abcam, UK). The chemiluminescence signal was developed using Amersham ECL Prime Western Blotting Detection Reagent following the manufacturer’s protocol (GE Healthcare Life Sciences) and captured by an Amersham Imager 600 Blot and Gel Imager using the automatic mode and a 30-sec exposure time.

### rOMV Imaging by Transmission Electron Microscopy (TEM)

A 10-μl droplet of diluted rOMV solution (1:5 in PBS) was placed on a 400-mesh copper grid and incubated for 10 min at room temperature. The grids were washed once with deionized water, and the samples were then stained with 2% uranyl acetate for 1 min. Excessive uranyl acetate was removed with filter paper. After grid desiccation, images of the OMVs were taken under a Hitachi HT7700 transmission electron microscope at Kasetsart University Research and Development Institute (KURDI, Kasetsart University, Thailand) at an accelerating voltage of 100 kV.

## Results

### Recombinant Plasmid Constructs Carrying *lipo-gfpmut2* for the Expression of Outer Membrane GFPmut2

The synthetic DNA fragments used in the current study were designed to include the restriction sites of NdeI at the 5′ end, BamHI at the junction between the lipoprotein signal peptide and the mature *gfpmut2* sequence, and XhoI at the 3′ end. NdeI and XhoI were included for introduction of the DNA insert into the expression vector, while BamHI was also available for future cloning purposes. pET-22b was chosen as the backbone for plasmid construction due to the presence of the NdeI and XhoI restriction sites at the very ends (5′ and 3′ ends respectively) of its original cloning site. A C-terminal polyhistidine tag was inserted right after the XhoI sequence to allow simple recombinant protein detection. GFP was chosen as a protein dummy in this proof-of-concept study for multiple reasons: (1) it is a well-studied reporter protein and its sequence has been thoroughly validated; (2) several mutated GFP variants for better detection and visualization of the protein are available for use in subsequent experiments; and (3) the expression of this protein and its variants is relatively easy to detect through several approaches, for example, fluorescence microscopy, UV light excitation, and antibody probing. This study adopted *gfpmut2* as the gene of interest.

**Fig. 1:**
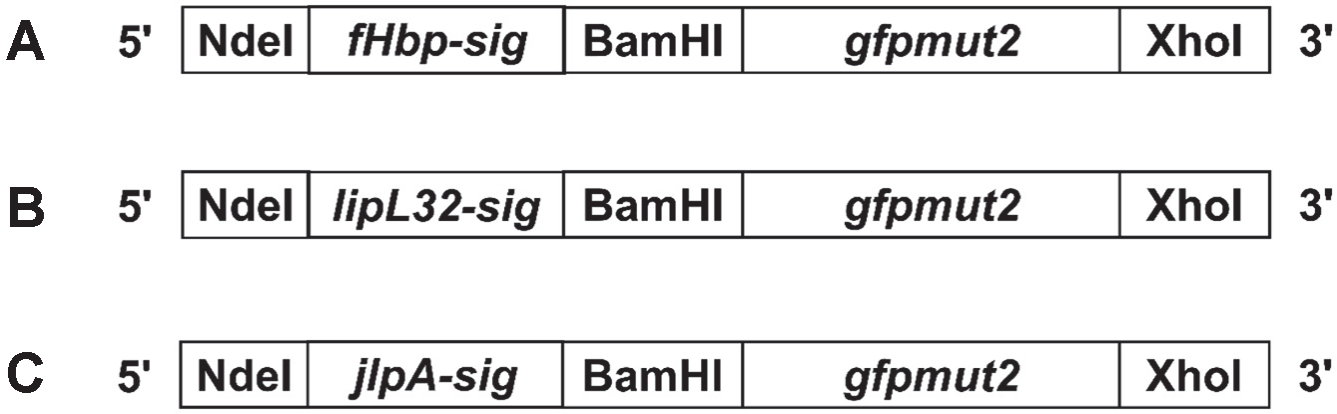
Schematic representation of heterologous *lipo-gfpmut2* constructs. (**A**) *lipo-fHpb-gfpmut2*; (**B**) *lipo-lipL32-gfpmut2*; (**C**) *lipo-jlpA-gfpmut2*; sig: signal peptide including tether sequence.

**Fig. 2:**
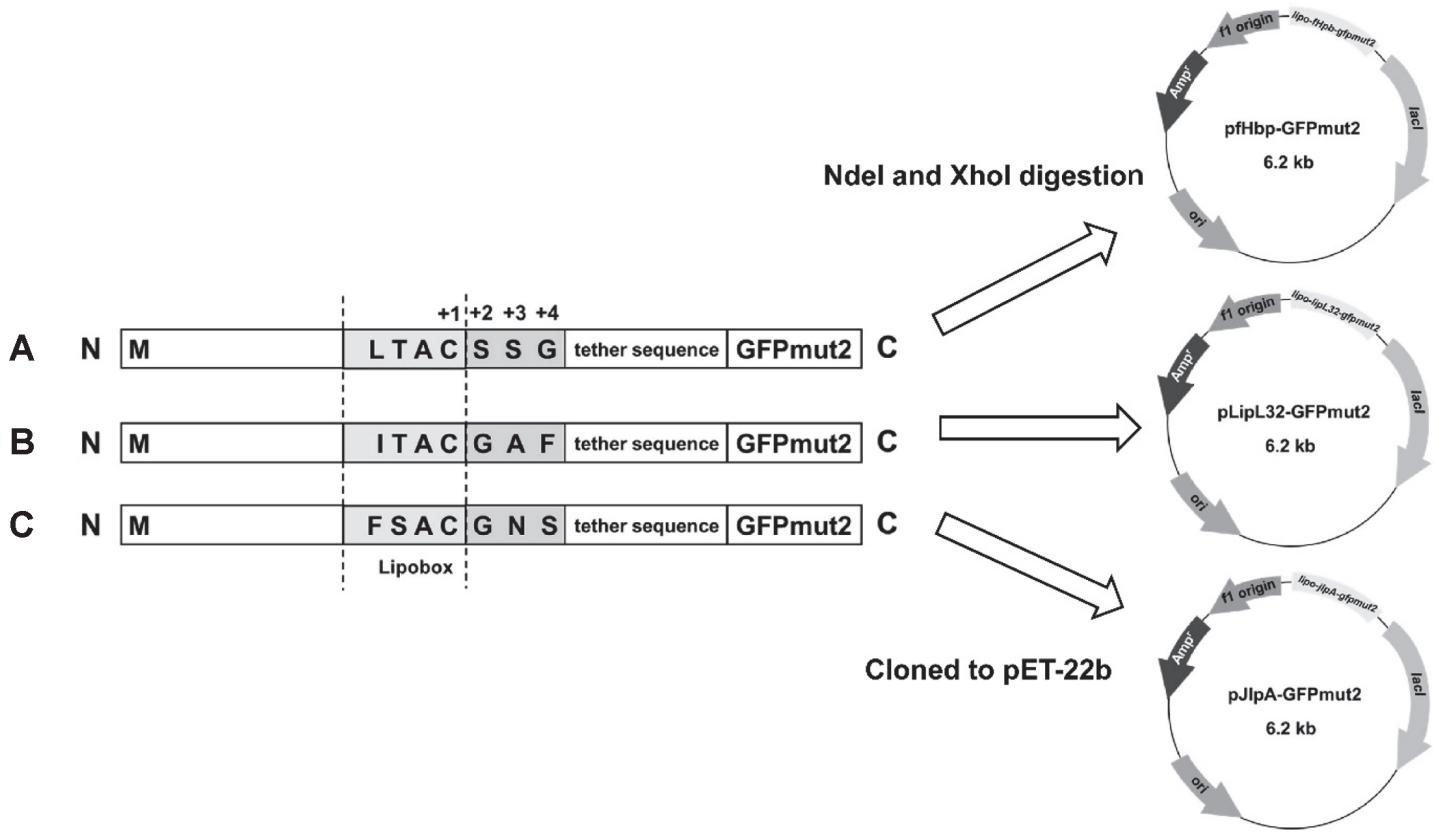
Amino acid sequences of the lipobox and Lol sorting signals at position +2, +3, and +4 of *lipo-gfpmut2* fragments and their corresponding recombinant plasmid constructs. (**A**) *lipo-fHpb-gfpmut2*; (**B**) *lipo-lipL32-gfpmut2*; (**C**) *lipo-jlpA-gfpmut2*.

Type II signal peptides including their Lol (lipoprotein outer membrane localization) sorting signals (amino acid at position +2, +3, and +4 after +1 cysteine residue of the conserved lipobox) and tether sequences of three well-characterized bacterial outer membrane lipoproteins, fHpb, LipL32, and JlpA, were merged upstream to the mature *gfpmut2* sequence. This process resulted in three chimeric *lipo-gfpmut2* fragments: *lipo-fHpb-gfpmut2*, *lipo-lipL32-gfpmut2*, and *lipo-jlpA-gfpmut2*, which were 834, 825, and 804 base pairs long, respectively (Figs. 1 and 2). Recombinant pET-22b plasmids carrying the *lipo-gfpmut2* fragments for outer membrane GFPmut2 expression were correspondingly termed pfHpb-GFPmut2, pLipL32-GFPmut2, and pJlpA-GFPmut2 (Fig. 2).

### Analysis and Expression of Heterologous GFPmut2

Putative amino acid sequences of *lipo-fHpb-gfpmut2*, *lipo-lipL32-gfpmut2*, and *lipo-jlpA-gfpmut2* were acquired by translating the nucleotide sequences using Translate, a free online tool in the ExPaSy Bioinformatics Resource Portal (https://web.expasy.org/translate/, accessed March 1, 2020). The presence of conserved lipobox and the subcellular location of the protein were predicted and are presented in Table 1. The theoretical molecular weight (MW) and isoelectric point (pI) of recombinant GFPmut2 (including the C-terminal polyhistidine tag) were determined by the Compute pI/MW tool in ExPaSY (https://web.expasy.org/compute_pi/, accessed March 1, 2020) (Table 2). Notably, the MW of GFPmut2 was calculated based on its mature protein sequence, in other words, the signal peptide was excluded from the calculation.

**Fig. 3:**
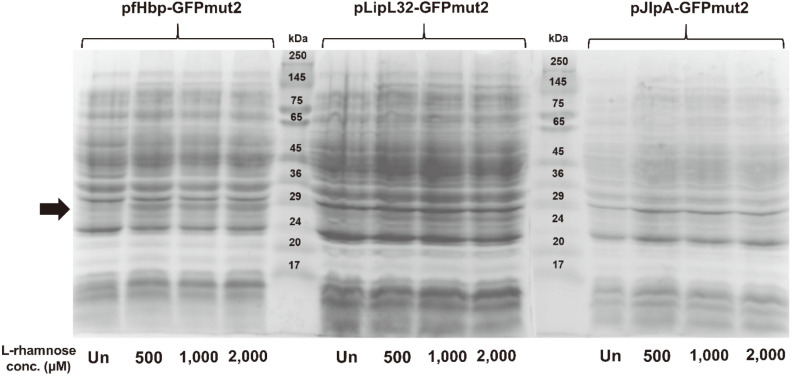
SDS-PAGE analysis of whole-cell lysate proteins from the pilot study. GFPmut2 protein expression was induced with 400 μM IPTG and varied concentrations of L-rhamnose (500, 1,000, and 2,000 μM). The calculated MW of the target protein is 30 kDa. Un: uninduced culture.

**Fig. 4:**
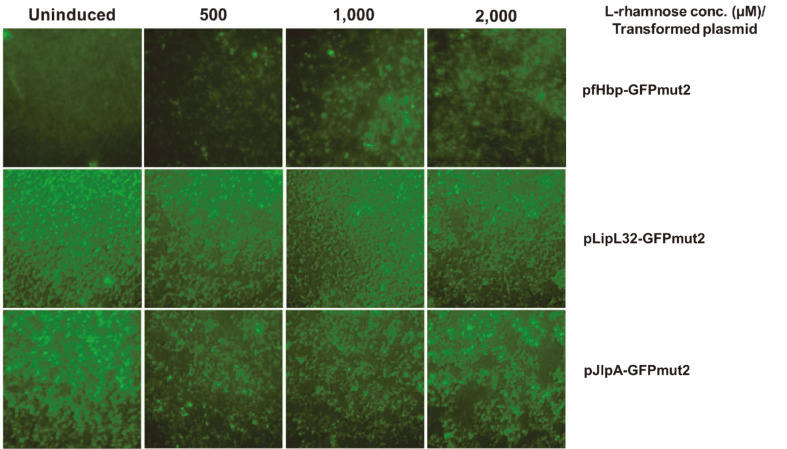
Fluorescence signal from Lemo21(DE3) cells. GFPmut2 expression in the pilot study was induced with different L-rhamnose concentrations.

SDS-PAGE analysis of the whole-cell lysate proteins obtained from the pfHpb-GFPmut2, pLipL32-GFPmut2, and pJlpA-GFPmut2 expression cultures in the pilot study revealed that one of the heterologous *lipo-gfpmut2* fragments cloned into the plasmids was able to be recognized by the bacterial gene expression machinery and could be translated. pfHpb-GFPmut2 was the best candidate for large-scale expression since the protein band at the expected size (approximately 30 kDa) in this expression culture showed the highest intensity among the group. In addition, no protein band was detected in the uninduced control, implying that the expression was tightly regulated. This was considered beneficial to the host cells because the overexpression of membrane proteins is usually toxic. Inducing expression with 2,000 μM L-rhamnose led to the highest level of protein production (Fig. 3). This was in accordance with the GFPmut2 fluorescence microscopy results, that is, the pfHpb-GFPmut2 expression culture had the strongest fluorescence emission when the lysate samples were excited at 488 nm (Fig. 4). Preliminary expression tests of pLipL32-GFPmut2 and pJlpA-GFPmut2 under the same conditions as pfHpb-GFPmut2 showed no detectable protein bands at the target size. Additionally, there were no differences in fluorescence among cells from the different L-rhamnose induction concentrations.

Therefore, only the pfHpb-GFPmut2 construct progressed to large-scale expression culture to produce recombinant OMVs. GFPmut2 production in Lemo21(DE3) cells was induced with 400 μM IPTG and 2,000 μM L-rhamnose. The total protein concentration of OMVs extracted from cell-free culture media was assessed with a DC protein assay and was found to be 4.5 μg/μl. Thus, the total OMV protein yield was approximately 4 mg/500 ml culture.

### Identification and Characterization of Heterologous GFPmut2 Incorporated into rOMVs

**Fig. 5:**
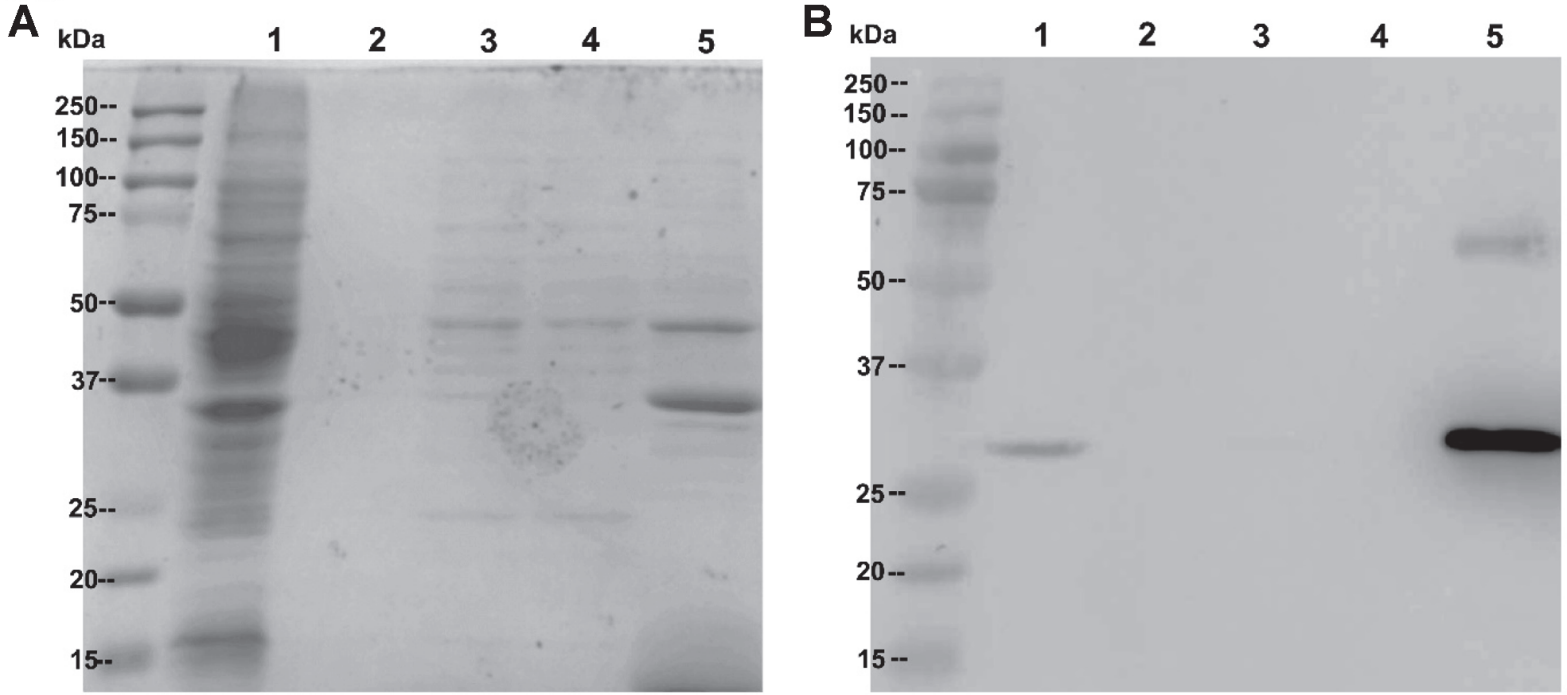
(**A**) SDS-PAGE and (**B**) Western blot analyses of total proteins from large-scale recombinant protein production. Lane 1: whole-cell lysate proteins from the pilot study, culture was induced with 2,000 μM L-rhamnose; 2: culture filtrate; 3: concentrated culture filtrate; 4: ultracentrifugation supernatant; 5: resuspended OMVs.

SDS-PAGE followed by Coomassie blue staining of total OMV proteins revealed a number of proteins with a wide molecular mass range (approximately 25 to 150 kDa). The most distinct band was located between 25 and 37 kDa (Fig. 5A, lane 5), which matched the putative size of GFPmut2 including the polyhistidine tag (30 kDa). Fig. 5B shows a strong chemiluminescence signal at the putative size of the target protein (between 25 and 37 kDa) in the Western blot of total OMV protein using anti-His mAb as the primary antibody. The same signal, but weaker, was also detected in the whole-cell lysate sample from the small-scale expression study under the same culture conditions. The strong intensity of the protein band seen in both the Coomassie blue-stained acrylamide gel and on the blotting membrane indicated successful overexpression of the target protein in this system.

### Visualization of rOMVs

**Fig. 6:**
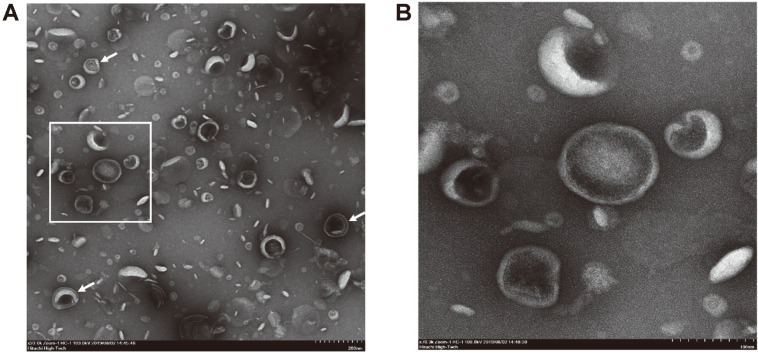
TEM images of the OMV pellets acquired by ultracentrifugation of concentrated culture media. (**A**) 20,000× magnification shows that the majority of rOMVs were hollow, single-membrane spherical structures. Arrows indicate vesicles encased in double membranes. (**B**) rOMVs at the higher magnification (inset).

Visualization of the rOMVs under TEM confirmed that the nanoparticles produced and extracted using the current platform were hollow, closed spherical structures with diameters varying from 20 to 200 nm. While most of the vesicles were enclosed by a single membrane corresponding to the bilayer outer membrane of the host cell and shared similar electron micrograph characteristics to previously examined vesicles [18, 19], a small number of vesicles were found to have double membranes. The exterior sheet stemmed from the outer membrane of the bacteria, and the interior lamina was derived from the cytoplasmic membrane (Fig. 6).

## Discussion

Over the past decade, OMVs from gram-negative bacteria, either in their native or detergent-extracted form, have been applied in vaccine development [20]. They have been used not only as antigen delivery vehicles to the host and as the antigens themselves but also as potential intrinsic vaccine adjuvants. This ability is due to the presence of multiple immunogenic molecules that can potentiate the innate immune response, leading to stronger specific immune responses toward the antigens of interest [5]. Owing to their immunological features and clinical success, especially in the case of Bexsero®, attempts have been made to develop new vaccines. However, some extraction methods lead to OMVs that contain lower amounts of possible immunogenic proteins when compared to OMVs that are spontaneously released [21]. Thus, studies have attempted to build systems to produce recombinant bioengineered OMVs that display the desired vaccine antigens to overcome this issue. These attempts have been carried out in multiple gram-negative bacteria, including *E. coli*, *N. meningitidis*, *N. flavescens*, *Vibrio cholerae*, and *Salmonella* Typhimurium [22-26].

In the current study, the key concept of platform development for rOMV production is to enable the *E. coli* bacterial host to recognize the heterologous sequence, particularly the lipoprotein signal peptide that is fused to the gene of interest. This recognition allows the cell to produce and transport the target protein across its inner membrane and relocate the protein to the outer membrane. Here, the target protein is likely to be incorporated into the vesicles that are formed and released into the culture media. This was indeed confirmed after the OMVs were isolated. To prove this concept, several lipoprotein signal peptides along with their tether sequences were combined with the mature sequence of the model protein, GFPmut2, and cloned into an expression vector, followed by transformation into the host cells. GFPmut2 expression was induced and the rOMVs containing the protein of interest were harvested. If this concept proves to be broadly attainable, any type of protein could be applied as the antigen, not only bacterial proteins. The protein of interest could be engineered in place of GFPmut2; therefore, the platform developed here could potentially become the backbone of OMV-based vaccine production. *E. coli* strain B was selected as the host for protein expression for several reasons: (1) it is a gram-negative bacterial species that is known for its ability to produce OMVs and is genetically well-characterized and widely used in biotechnology; (2) numerous protein expression systems compatible with this species are available; and (3) it is relatively easy to manage, providing a reasonably low cost of production upon further scale-up.

After the target gene is translated to protein, Sec translocation machinery of the bacteria recognize the lipoprotein signal peptide at the N terminus of the recombinant protein (in the form of lipoprotein precursor) and transport the protein across cytoplasmic membrane to the periplasmic surface [27]. At this location, the precursor protein is lipid-modified at the +1 cysteine residue of the conserved lipobox and becomes a mature lipoprotein through the action of several enzymes (preprolipoprotein diacylglyceryl transferase, lipoprotein signal peptidase, and apolipoprotein N-acyltransferase) [28]. Due to the absence of aspartate amino acid at position +2, a so-called Lol avoidance signal, the mature lipidated target protein is localized to the outer membrane by the Lol pathway proteins [29] and is likely to be included in the OMVs. This is why three different signal peptides were selected for investigation, two from surface-exposed gram-negative bacterial lipoproteins and one from a subsurface spirochetal lipoprotein.

Of the three constructs used in this study, the plasmid carrying *lipo-fHpb-gfpmut2* fragment (a fusion between N-terminal sequence of *N. meningitidis* fHbp and GFPmut2) was the only construct that successfully expressed the *gfpmut2* sequence and the protein could be relocated to the outer membrane of the host cells. GFPmut2 protein was detected in the vesicles isolated from the culture media without the application of any physical or chemical disruption. This implied that the engineered heterologous sequence was effectively recognized by the expression, secretion, and Lol machineries of the host cells. These processes resulted in the production and transportation of recombinant protein to the outer membrane, as the rOMVs retrieved in this study were sOMVs, which result from the autonomous, spontaneous bulging and blebbing of the outer membrane compartment. Moreover, because there was no physical or chemical cell disruption, it is unlikely that the vesicles would involve components from the inner membrane. Previous attempts were made to create surface-exposed antigens by combining the heterologous antigen of interest with *fHbp* sequence. In that experiment, the N-terminal portion of fHbp (the signal peptide and tether sequence, amino acid positions 1–33) was required for the expression and relocation of *Borrelia burgdoferi* OspA antigen to be surface-displayed in the *N. meningitidis* host and on meningococcal OMVs [23]. The results of the present study confirmed the ability of fHbp signal peptide in targeting the desired protein to the outer membrane of the host, and thus to the OMVs in different expression host species.

**Fig. 7:**
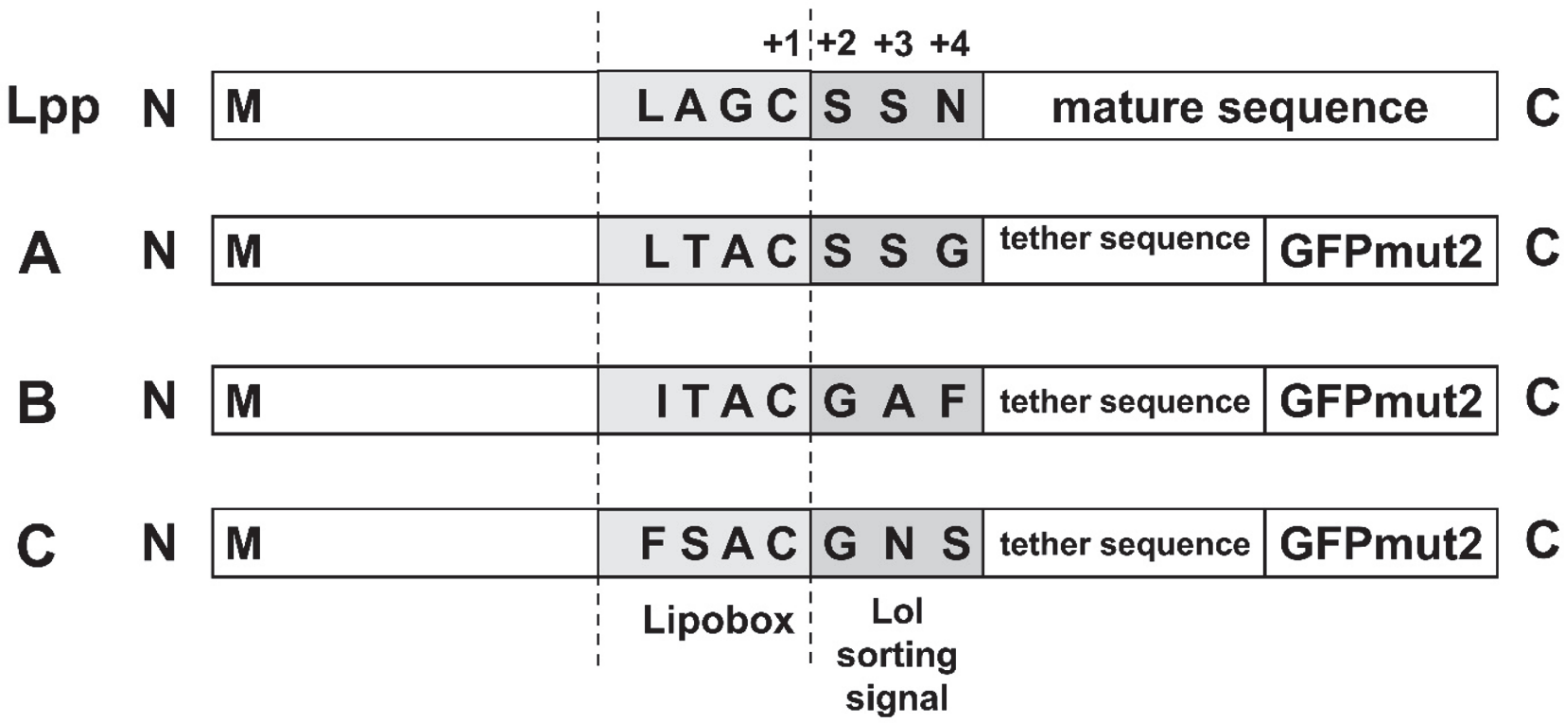
Comparison of lipobox sequences and Lol sorting signals between an *E. coli* major outer membrane lipoprotein, Lpp, and the 3 heterologous *lipo-gfpmut2* constructs. (**A**) *lipo-fHpb-gfpmut2*; (**B**) *lipo-lipL32-gfpmut2*; (**C**) *lipo-jlpA-gfpmut2*.

When all three lipoprotein signal peptides including their amino acids at position +2 and +3 were compared to those of Lpp protein, the major outer membrane lipoprotein of *E. coli*, a higher degree of sequence dissimilarity (especially at the conserved lipobox) was observed for *lipo-lipL32-gfpmut2* and *lipo-jlpA-gfpmut2* fragments (Fig. 7). This may explain why the use of LipL32 and JlpA signal peptides were not successful in directing the target protein to the host outer membrane. On the contrary, the higher degree of similarity for fHbp signal sequence could explain why the host machinery was able to recognize the fused sequence and properly process the target protein; the lipobox sequences and the signals for the Lol pathway machinery of fHbp and Lpp proteins are highly similar.

In conclusion, this study provided evidence that the lipoprotein signal peptide of fHbp, a surfaced-exposed lipoprotein of *N. meningitidis*, could be used as a functional signal to direct the heterologous protein, GFPmut2, to the outer membrane of *E. coli*, resulting in packaging of the protein into the vesicles. Although this is a proof-of-concept study of a prototype platform for the production of rOMVs and is not yet optimized, we believe that it has strong potential to be developed as an OMV-based vaccine platform and as nanomolecule delivery vehicles. Future studies should replace the model protein with an antigen of interest and investigate the yield, stability, and uniformity of the rOMVs, as well as the immune response toward these nanoparticles.

## Figures and Tables

**Table 1:** SignalP prediction of recombinant GFPmut2 subcellular location corresponding to *lipo-gpfmut2* fragments with different signal peptide sequences.

*lipo-gpfmut2* fragment	Length of signal peptide (aa)	Type of signal peptide	Probability	Cleavage site (aa position and sequence)
*lipo-fHpb-gfpmut2*	21	Lipoprotein (Sec/SPII)	0.9940	20-21, LTA-CS
*lipo-lipL32-gfpmut2*	21	Lipoprotein (Sec/SPII)	0.9893	20-21, ITA-CG
*lipo-jlpA-gfpmut2*	19	Lipoprotein (Sec/SPII)	0.9997	18-19, FSA-CG

aa: amino acid; Sec/SPII: type II or lipoprotein signal peptide.

**Table 2:** Characterization of recombinant GFPmut2 expressed by the three recombinant plasmid constructs.

Plasmid name	Length of target protein (aa)^a^	MW (kDa)	pI	MW of lipid moiety (kDa)	Estimated total MW (kDa)
pfHpb-GFPmut2	264	29.4	5.86	0.7	30.1
pLipL32-GFPmut2	261	29.3	6.04	0.7	30
pJlpA-GFPmut2	256	28.9	5.86	0.7	29.6

aa: amino acid; ^a^: includes C-terminal 6xHis tag with exclusion of N-terminal lipoprotein signal peptide; MW: molecular weight; kDa: kilodalton; pI: isoelectric point.
